# Exosomal hsa_circRNA_104484 and hsa_circRNA_104670 may serve as potential novel biomarkers and therapeutic targets for sepsis

**DOI:** 10.1038/s41598-021-93246-0

**Published:** 2021-07-08

**Authors:** Chang Tian, Jiaying Liu, Xin Di, Shan Cong, Min Zhao, Ke Wang

**Affiliations:** Department of Respiratory Medicine, The Second Hospital of Jilin University, Changchun, Jilin China

**Keywords:** Biomarkers, Medical research, Pathogenesis

## Abstract

In order to explore the role of exosomal circRNAs in the occurrence and development of sepsis, we looked for potential diagnostic markers to accurately identify sepsis and to lay a molecular basis for precise treatment. Ultracentrifugation was used to extract exosomes from the serum of patients with sepsis and healthy individuals. Then, changes in circRNA expression in exosomes were studied by circRNA microarray analysis. Gene ontology (GO) analysis and Kyoto City Encyclopaedia of Genes and Genomes (KEGG) pathway analysis were used to annotate the biological functions and pathways of genes, and a circRNA-miRNA-mRNA regulatory network was constructed. In the microarray analysis, 132 circRNAs were significantly differentially expressed, including 80 and 52 that were upregulated and downregulated, respectively. RT-qPCR verified the results of microarray analysis: hsa_circRNA_104484 and hsa_circRNA_104670 were upregulated in sepsis serum exosomes. ROC analysis showed that hsa_circRNA_104484 and hsa_circRNA_104670 in serum exosomes have the potential to be used as diagnostic markers for sepsis. The circRNA-miRNA-mRNA network predicted the potential regulatory pathways of differentially expressed circRNAs. There are differences in the expression of circRNA in serum exosomes between patients with sepsis and healthy individuals, which may be involved in the occurrence and development of the disease. Among them, elevations in hsa_circRNA_104484 and hsa_circRNA_104670 could be used as novel diagnostic biomarkers and molecular therapeutic targets.

## Introduction

Sepsis is defined as life-threatening organ dysfunction and is not a specific disease, but rather a syndrome of physiological, pathological, and biochemical abnormalities caused by the host's unregulated response to infection^1^. Sepsis is a heterogeneous disease state that progresses rapidly, and its early diagnosis and intervention can significantly improve prognosis^2^. Our diagnosis of sepsis mainly relies on Sequential Organ Failure Assessment (SOFA) scoring system, which has certain limitations; currently, there is no ‘gold standard’ for laboratory diagnosis. With the development of high-throughput sequencing technology, genomics and metabolomics analyses have found that the levels of various genes and metabolites in sepsis have changed, and that the changes occur earlier than clinical symptoms^3,4^. Identifying these molecular changes in sepsis is highly valuable for understanding the course of the disease, and for predicting prognosis and response to treatment. Exploring the changes in sepsis at cellular and molecular levels is helpful to explore the nature of its pathogenesis and may help to identify the causes of heterogeneity in the body's response^5^. Individualised therapy targeting the core molecules of the disease can improve the efficiency of the treatment and reduce toxicity. Therefore, these differentially expressed molecules may serve as diagnostic markers for sepsis and may become targets for molecular targeted therapy.

Exosomes are small extracellular vesicles derived from the endosomal system, ranging from 40 to 160 nm (about 100 nm on average) in diameter^6^. In sepsis, exosomes are secreted by a variety of cells (including mesenchymal stem cells and macrophages, among others), and act on recipient cells (e.g., cardiomyocytes, macrophages, vascular endothelial cells) to promote inflammation, inhibit inflammation, or regulate immunity^7–9^. Their contents are rich and diverse, containing a variety of proteins, DNA, RNA (e.g., mRNA, miRNA, lncRNA, circRNA), amino acids, and metabolites^6^. The uptake of cytoplasmic components during exosomal biogenesis is not random, but is a highly regulated and selective process, which is very important for disease identification and diagnosis^10^. The cell-free RNA in the blood is easily inactivated by endogenous RNase, while RNA encapsulated in exosomes can be prevented from degradation by RNase and can exist stably^11^. In addition, the exosomes released to the outside of cells exist in a variety of body fluids and are easy to separate and extract^11,12^. These characteristics give exosomes diagnostic and therapeutic potential.

CircRNA is a large class of non-coding RNAs produced by reverse splicing events^13^. CircRNAs are produced in the nucleus and are then transported to the cytoplasm. They have the characteristics of tissue specificity, cell specificity, high stability, and species conservation^14^. Some can be distributed to exosomes, where they are enriched and stably exist^15,16^. In disease states, the expression level of exosome circRNA changes, and it plays a regulatory role in cell proliferation, tumour metastasis, and drug resistance, among other processes^17^. CircRNAs are involved in the occurrence and progression of various diseases through multiple mechanisms. For example, circRNAs act as miRNA sponges to regulate gene expression and participate in the occurrence and development of tumours^13^; they also act as a protein sponge to mediate the immune response during viral infection^18^.

Numerous studies have shown that the expression of exosomal circRNAs is different between patients and healthy people, and its detection can help to identify patients. Therefore, exosomal circRNAs may be used as novel disease diagnostic markers^19^. To date, there have been no reports on the expression or role of exosomal circRNAs in sepsis. This study aimed to detect circRNAs in serum exosomes of patients with sepsis and to explore their value in the diagnosis of sepsis and in molecular targeted therapy.

## Materials and methods

### Patient samples and ethics statement

In this study, a total of 25 patients with sepsis who underwent treatment at the Second Hospital of Jilin University from September 2018 to January 2019 were included, in addition to 22 healthy individuals. Sepsis was defined according to the Sepsis-3 criteria^1^. All study participant’s peripheral blood samples (4–5 mL) were collected in the early phase (within 24 h) of the diagnosis of sepsis and centrifuged at 3000 rpm for 10 min to obtain the serum, which was stored at − 80 °C after being labelled. The patients’ clinical and laboratory data are shown in Table 1. This study was approved by the Ethics Committee of the Second Hospital of Jilin University. All experiments were performed in accordance with relevant named guidelines and regulations. All participants signed an informed consent form.

**Table 1:** Demographic characteristics of septic patients.

Characteristics	Septic patients (N = 22)
**Sex**	
Male, n (%)	16 (73)
Female, n (%)	6 (27)
Age, years	56.73 ± 16.12
Mortality, n (%)	8 (36)
**Comorbidities**	
Hypertension, n (%)	10 (45)
Diabetes, n (%)	7 (32)
**Source of sepsis**	
Abdominal, n (%)	3 (14)
Lung, n (%)	19 (86)
Mean arterial pressure, mmHg	91.509 ± 10.6399
PaO_2_/FiO_2_ (mmHg)	200.535 ± 78.7067
Use of mechanical ventilation, n (%)	5 (23)
**Hematologic and inflammatory data**	
Leukocyte, 10^9^/L	11.20 (8.75–14.05)
Neutrophils, 10^9^/L	9.60 (6.60–11.59)
Hemoglobin, g/dL	115.091 ± 21.5338
Platelets, 10^9^/L	128.282 ± 82.8287
Procalcitonin, ng/mL	7.69 (2.20–24.31)
SOFA score	6.273 ± 2.9469
Positive blood culture	5 (23)

Data are expressed as number (%), mean ± SD, or median (25th–75th percentile).

### Exosome collection

We used ultracentrifugation to extract exosomes from the serum, and the whole process was completed at 4 °C. First, the serum was centrifuged at 2000 × *g* for 30 min to remove dead cells and was then centrifuged at 10,000 × *g* for 30 min to remove cell debris and impurities. Then, the exosomes were preliminarily precipitated by centrifugation at 110,000 × *g* for 80 min. Phosphate buffer saline (PBS) solution was added to wash the soluble protein impurities, and then the sample was centrifuged again at 110,000 × *g* for 80 min to obtain pure exosomes. Finally, the pellet was resuspended in PBS solution (100μL PBS solution per 1 mL of serum) and was stored in a − 80 °C freezer.

### Western blotting analysis

Exosomal marker proteins were detected by immunoblotting. Protein was extracted from the same volume of exosomes, and the protein concentration of exosomes was quantified using the BCA method (Beyotime, China). Then, 20 μg of exosomal protein was separated by electrophoresis on a 12% SDS-PAGE gel and was then transferred to a PVDF membrane (Millipore, USA). Immunoblotting was performed with anti-CD63 and anti-TSG101 antibodies (Affinity, USA) at 4 °C. The primary antibodies were then detected with a horseradish peroxidase-conjugated secondary antibody (#SA00001-1 or #SA00001-2; Proteintech Group, USA). Finally, the ECL chemiluminescence agent (Thermo Fisher Scientific, USA) was used to display protein bands, and the results were recorded with photos.

### Electron microscopy

For electron microscopy, 5 μl of the exosome suspension was spotted on copper mesh and dried at room temperature. The sample was then negatively stained with 5 μl of 2% (w/v) phosphotungstic acid solution. The morphology of exosomes was observed at 80 kV under a transmission electron microscope (JEM-1400, JEOL, Japan), and the results were photographed.

### RNA extraction and quality control

Total RNA was extracted from the exosome suspension using the TRI Reagent BD (Molecular Research Center, Inc., USA) according to the manufacturer’s protocol. The total RNA from each exosome sample was quantified and its purity was evaluated using a NanoDrop 2000 ultra-micro spectrophotometer (Thermo Fisher Scientific, USA).

### circRNA microarray analysis

CircRNA microarray analysis was performed on serum exosomes from three people with sepsis and three healthy persons. According to the manufacturer's protocol (Arraystar Inc., USA), sample labelling and microarray hybridization were performed. First, RNA was fluorescently labelled. Rnase R reagent (Epicenter, Inc., USA) was used to digest total RNA to remove linear RNA and enrich circRNAs. The enriched circRNAs were then transcribed into fluorescently labelled cRNA using a random priming method (Arraystar Super RNA Labelling Kit; Arraystar, USA). The labelled cRNAs were purified using the RNeasy Mini Kit (Qiagen, Germany). Microarray hybridisation was then performed in an Agilent Hybridisation oven. The fluorescently labelled cRNAs were cleaved into fragments and were then hybridised on the circRNA expression microarray slide. After hybridisation was completed, the hybridised microarrays were washed, fixed, and scanned using the Agilent Scanner G2505C. Agilent Feature Extraction software was used to extract raw data from the scanned images. Quantile normalisation of raw data was performed using the limma package (version 3.48.0)^20^ in R, and the circRNAs labelled by the software were retained for subsequent difference analysis. A *t*-test was used to estimate the statistical significance of the difference. Fold changes and *p*-values were used to screen for significant differences in the expression of circRNAs between the two groups of samples. Volcano plots and heat maps were used to display differentially expressed circRNAs.

### real-time quantitative PCR (RT-qPCR) analysis

Total RNA was extracted from serum exosomes of 25 sepsis patients and 22 controls. Real-time quantitative polymerase chain reaction (RT-qPCR) was used to verify the experiment. The sequences of the primers used in the experiment are shown in Table 2. Total RNA was reverse transcribed into complementary DNA (cDNA) using a PrimeScript RT reagent kit (Takara, Japan) according to the manufacturer’s protocol. Real-time quantitative PCR reactions were then carried out with a real-time PCR system (LightCycler480, Roche, Switzerland) using TB Green Premix Ex Taq II (Takara, Japan). The PCR conditions were 95 °C for 30 s, followed by 40 cycles at 95 °C for 10 s, and 60 °C for 60 s. β-actin was used as a reference gene, and all qPCR reactions were repeated three times. The 2^-△△CT^ value reflects the relative expression level of circRNAs.

**Table 2:** Primers designed for qRT-PCR analysis of circRNAs.

Target ID	Primer sequence, 5’–3’	Tm (°C)	Product size in bp
β-actin (human)	F:5' GTGGCCGAGGACTTTGATTG3'	60	73
	R:5' CCTGTAACAACGCATCTCATATT3’		
hsa_circRNA_104484	F:5’ TGTATTCTCTCTGTGTGTGGCTG 3’	60	134
	R:5’ GCAACATCCCAAATCGGTCT 3’		
hsa_circRNA_104670	F:5’ CGCAGAAGCGTTGTCACTG 3’	60	110
	R:5’ CTTCCCCGTGTTCTTCCTGTT 3’		
hsa_circRNA_101491	F:5’ AGGCTTTTGGACAAGTGGGTG 3’	60	83
	R:5’TGAGGATGTGGTGCTGTTTGTG3’		
hsa_circRNA_406194	F:5’ ACAATGATGAGGCCTTAGAAGC 3’	60	58
	R:5’ CGATGGCATTCACCCTCTT 3’		
hsa_circRNA_103864	F:5’ GGATGTATGGTGTAGGTGTGGA 3’	60	90
	R:5’CAAGACTATTATCCTTTATTATAACCC3’		

### Functional analysis

Arraystar microRNA prediction software was used to predict miRNAs downstream of differentially expressed circRNAs. Then, the interactions between circRNA-microRNAs are explained in detail. TargetScan (http://www.targetscan.org/vert_71/), miRDB (http://www.mirdb.org/), and miRTarBase (http://mirtarbase.mbc.nctu.edu.tw/php/index.php) were used to predict the potential targets of miRNAs. The common genes in the three databases were collected using Venn diagrams. The circRNA–miRNA–mRNA regulatory map was visualised using Cytoscape 3.8.0. Gene ontology (GO) analysis was used to annotate the biological functions of genes in the ceRNA network, including molecular functions (MF), biological pathways (BP), and cellular components (CC). Kyoto City Encyclopaedia of Genes and Genomes (KEGG) Enrichment Analysis was used to evaluate the biological pathways of genes^21^. The enrichment of MF, BP, CC, and pathways of genes were annotated with DAVID 6.8 (https://david.ncifcrf.gov/) which is an online biological tool.

### Statistical analysis

SPSS software (version 23.0, IBM, Chicago, IL, USA) was used for statistical analysis. If the data of continuous variables were distributed normally, the data were analysed using *t*-tests; results are expressed as the mean ± standard deviation. If data were non-normal, the Mann–Whitney U test was used, and the data are expressed in percentile form. Data of categorical variables between groups were tested using the Chi-square test. A *p* value of < 0.05 means that the difference is statistically significant. The receiver operating characteristic (ROC) curve was constructed to evaluate the diagnostic ability of exosomal circRNAs for sepsis. The area under the ROC curve (AUC) was used to evaluate the diagnostic efficacy of circRNA. The Youden Index was used to determine the optimal cut-off value, sensitivity and specificity (Youden Index = Sensitivity + Specificity-1). The highest Youden index corresponds to the optimal cut-off value, sensitivity and specificity.

### Ethics approval and consent to participate

This study was approved by the Ethics Committee of the second hospital of Jilin University. All participants were informed and willing to sign informed consent.

### Consent for publication

All the authors read and consented to the publication of the manuscript.

## Results

### Characterization of circulating serum exosomes

**Figure 1:**
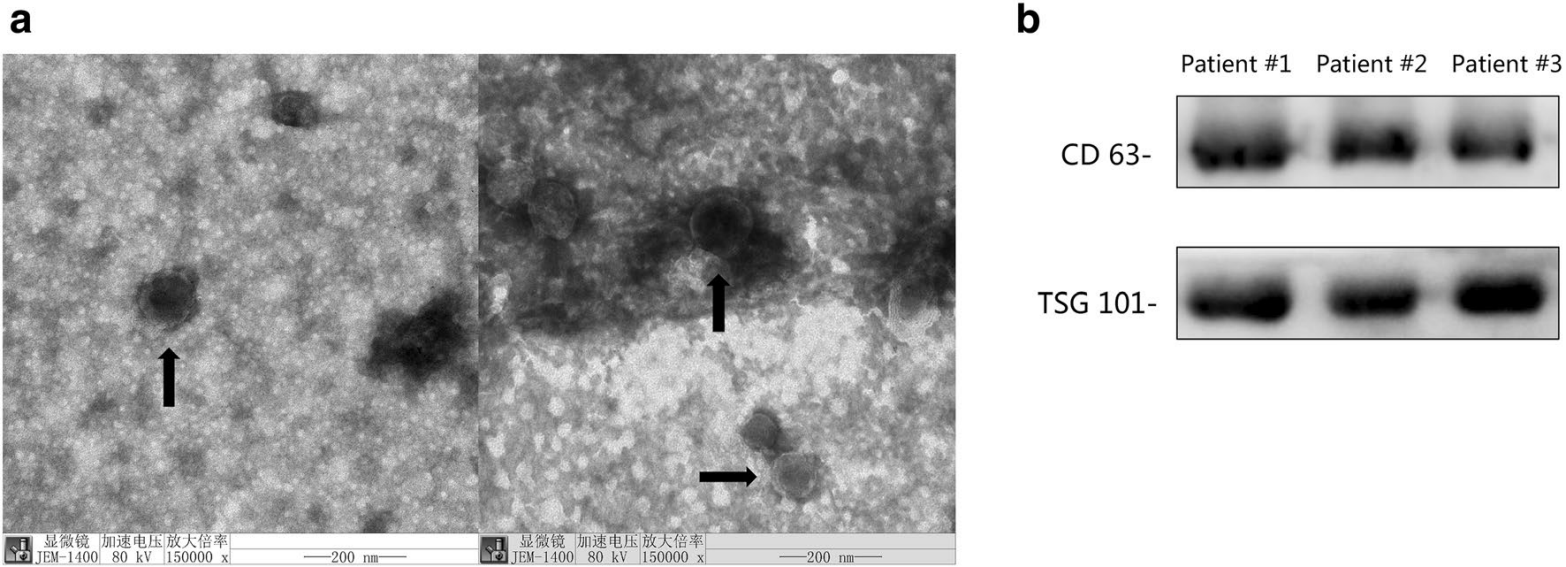
(**a**) Electron micrographs and (**b**) WB results of serum exosomes.

The serum exosome was confirmed by transmission electron microscopy (TEM) and WB for CD63 or TSG101 (Fig. 1a). The exosomes are round or oval ‘cup-shaped’, with a diameter in the range of 40–160 nm. CD63 and TSG101 showed positive expression in WB (Fig. 1b).

### Identification of differentially expressed circRNAs

**Figure 2:**
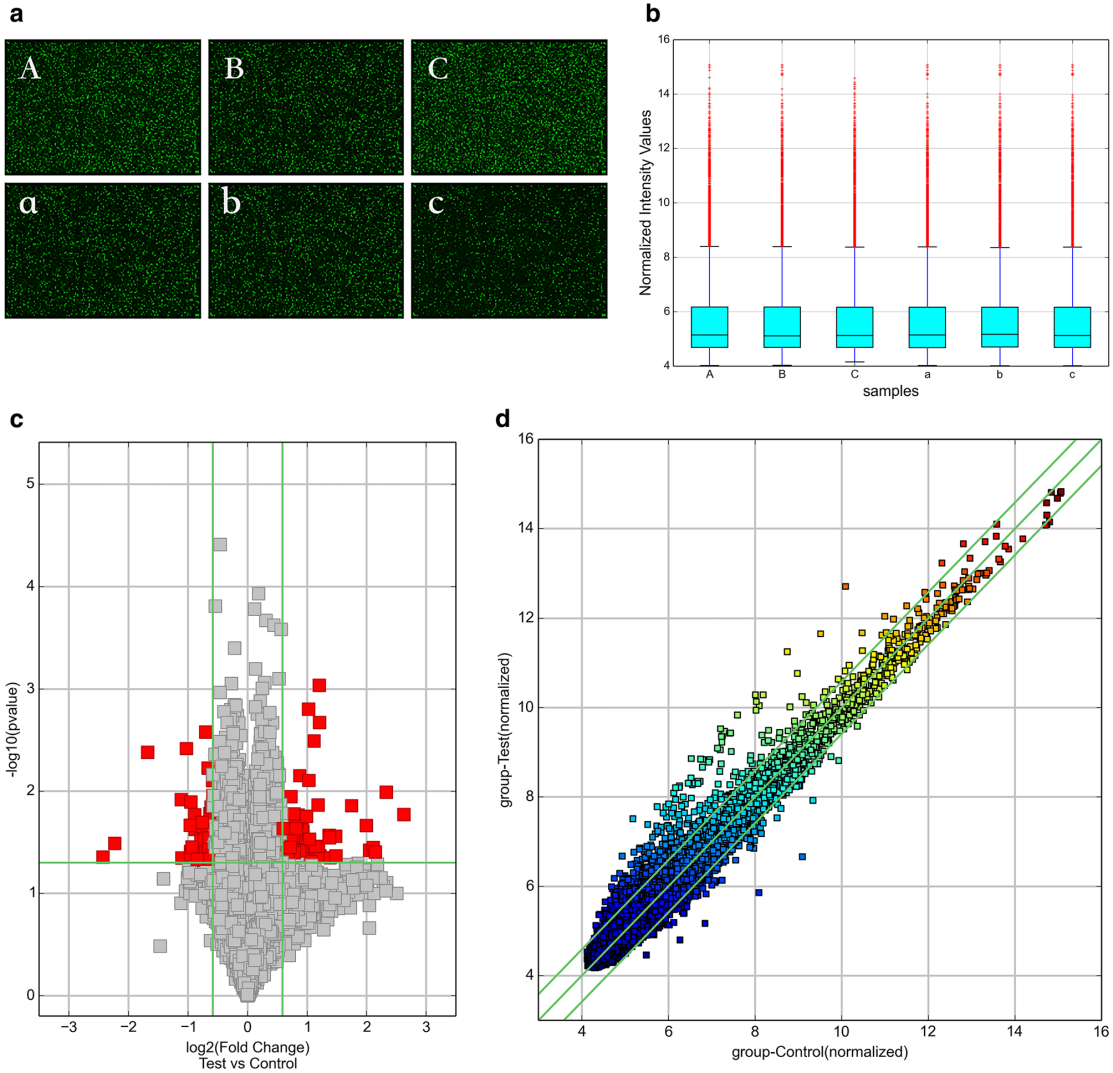

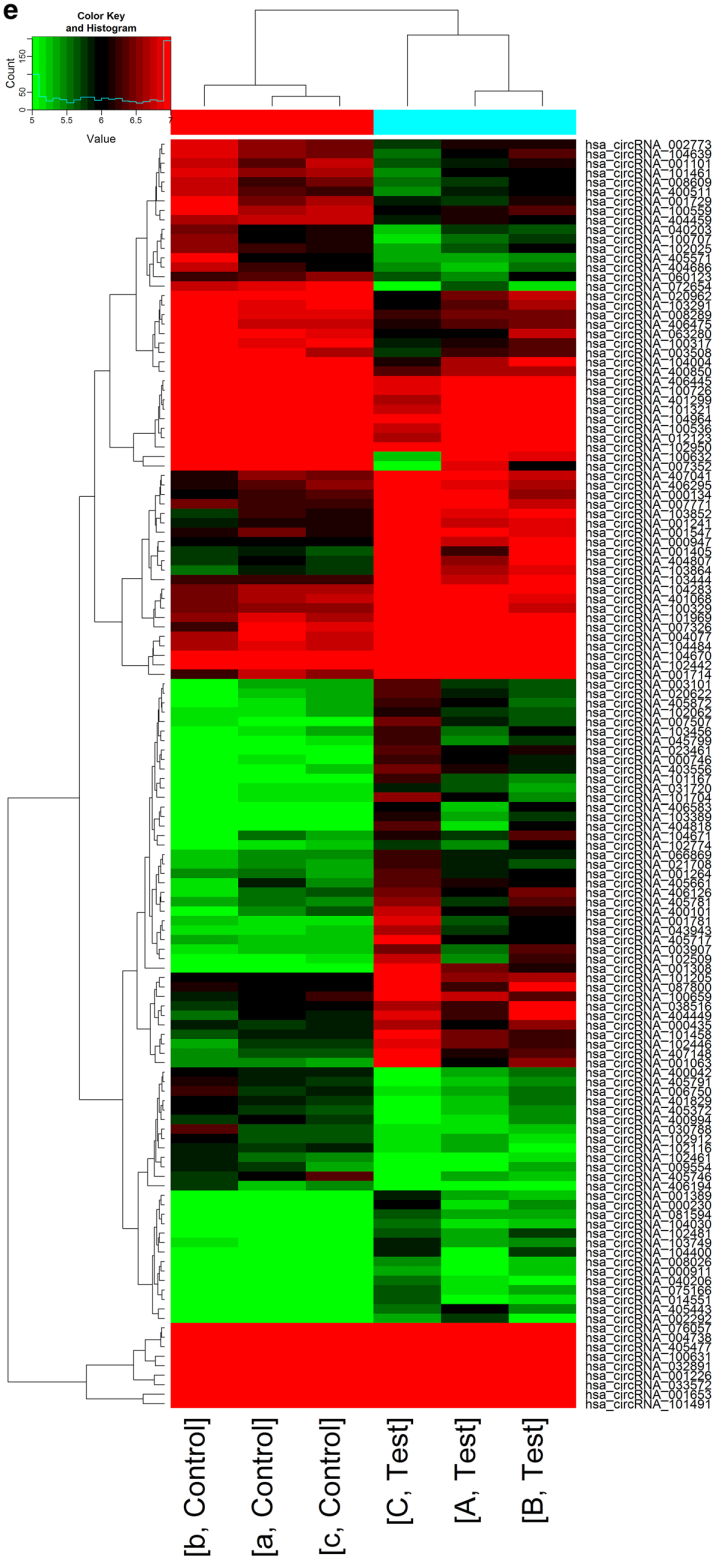
(**a**) The probe fluorescence signal displayed in each microarray scanning picture was uniform and clear. (A, B, C: sepsis group, a, b, c: control group). (**b**) Box plot: The abscissa represents each sample, and the ordinate represents the normalized intensity value. The expression of circRNAs in each sample was almost the same after normalization. (**c**) Volcano map: Differentially expressed circRNAs between sepsis and healthy human serum exosomes. (**d**) Scatter plot: Changes of circRNAs expression levels between sepsis and healthy human serum exosomes. (**e**) Cluster analysis: the distinguishable circRNA expression profile between sepsis and healthy human serum exosomes. The quantile normalisation and difference analysis were performed using limma package (version 3.48.0) in R. The Volcano map and Scatter plot were performed using python (version 2.7). Cluster analysis was performed using gplots package (version 3.1.1) in R.

We used circRNA microarray technology to detect changes in the circRNA expression profile of serum exosomes in sepsis. After scanning the fluorescent signal of circRNA microarray hybridisation, a total of six scanning pictures of the sepsis and control groups were obtained (Fig. 2a). The box plot shows the results of the quality control analysis of the microarray data (Fig. 2b). Volcano plots and scatter plots were used to visually show the differences in circRNA expression between the two groups. In the volcano map (Fig. 2c), the vertical lines represent 1.5 times up and down, and the horizontal lines represent *p* ≤ 0.05. Red dots indicate circRNAs that are significantly differently expressed, and grey dots indicate circRNAs that are not significantly differently expressed. In the scatter plot (Fig. 2d), the X-axis and Y-axis represent the normalised signal values of the two groups of samples, respectively, and the green line is the fold line. Plots distributed above the upper green line and below the lower green line represent significantly differently expressed circRNAs.

A total of 13228 circRNAs were detected by circRNA microarray analysis, of which 6247 were upregulated and 6981 were downregulated. Among them, 132 circRNAs were differentially expressed (*p* < 0.05, fold change > 1.5), including 80 upregulated and 52 downregulated circRNAs. Specific details are shown in Tables 3 and 4. Then, cluster analysis was performed on the significantly differentially expressed circRNAs to visually display the differentially expressed circRNAs and to test their rationality and accuracy. As shown in the heat map (Fig. 2e), red represents highly expressed circRNAs and green represents low-expressed circRNAs. The results showed distinguishable circRNA expression profiles between the two groups of samples.

**Table 3:** Differentially up-regulated circRNAs in serum exosomes of patients with sepsis.

circRNA	Alias	P-value	FDR	FC (abs)	chrom	circRNA_type	best_transcript	GeneSymbol
hsa_circRNA_066869	hsa_circ_0066869	0.022756307	0.431741635	1.5009586	chr3	Sense overlapping	NM_018266	TMEM39A
hsa_circRNA_405661		0.039569341	0.431741635	1.6109265	chr18	Sense overlapping	NR_033354	ZNF519
hsa_circRNA_001264	hsa_circ_0000086	0.017699179	0.431741635	1.5018914	chr1	Antisense	NM_152996	ST6GALNAC3
hsa_circRNA_104400	hsa_circ_0006944	0.043350982	0.431741635	1.7182999	chr7	Exonic	NM_001518	GTF2I
hsa_circRNA_101167	hsa_circ_0005916	0.024804956	0.431741635	1.9019977	chr12	Exonic	NM_012174	FBXW8
hsa_circRNA_407041		0.049595412	0.431741635	1.6179568	chr8	Sense overlapping	ENST00000518026	MSR1
hsa_circRNA_014551	hsa_circ_0014551	0.030830215	0.431741635	1.6101319	chr1	Exonic	NM_018489	ASH1L
hsa_circRNA_407148		0.024995712	0.431741635	1.839682	chr9	Intergenic		
hsa_circRNA_003101	hsa_circ_0003101	0.042393826	0.431741635	1.6219639	chr3	Exonic	NM_173471	SLC25A26
hsa_circRNA_033572	hsa_circ_0033572	0.007038362	0.431741635	1.8332939	chr14	Exonic	NM_138420	AHNAK2
hsa_circRNA_103389	hsa_circ_0001309	0.026886598	0.431741635	1.7955397	chr3	Exonic	NM_003157	NEK4
hsa_circRNA_401068		0.049692498	0.431741635	1.5372069	chr12	Exonic	NM_032814	RNFT2
hsa_circRNA_081594	hsa_circ_0081594	0.033763521	0.431741635	1.5187091	chr7	Exonic	NM_016068	FIS1
hsa_circRNA_104030	hsa_circ_0001564	0.026931184	0.431741635	1.5017159	chr5	Exonic	NM_001746	CANX
hsa_circRNA_104283	hsa_circ_0001667	0.027324991	0.431741635	1.7455824	chr7	Exonic	NM_017802	DNAAF5
hsa_circRNA_021708	hsa_circ_0021708	0.035339271	0.431741635	1.5242451	chr11	Exonic	NM_003477	PDHX
hsa_circRNA_103749	hsa_circ_0005480	0.041431749	0.431741635	1.5968689	chr4	Exonic	NR_036614	DCLK2
hsa_circRNA_008026	hsa_circ_0008026	0.025086019	0.431741635	1.5726361	chr4	Exonic	NM_001221	CAMK2D
hsa_circRNA_101205	hsa_circ_0006078	0.048933628	0.431741635	1.7621779	chr12	Exonic	NM_023928	AACS
hsa_circRNA_007507	hsa_circ_0007507	0.023237468	0.431741635	1.8572626	chr5	Exonic	NM_002890	RASA1
hsa_circRNA_103456	hsa_circ_0067127	0.027006569	0.431741635	1.6842385	chr3	Exonic	NM_012190	ALDH1L1
hsa_circRNA_031720	hsa_circ_0031720	0.04767514	0.431741635	1.5353758	chr14	Exonic	NM_006364	SEC23A
hsa_circRNA_075166	hsa_circ_0075166	0.025125707	0.431741635	1.5415749	chr5	Exonic	NM_022455	NSD1
hsa_circRNA_001781	hsa_circ_0001781	0.048181011	0.431741635	1.9555457	chr8	Intronic	ENST00000517494	CSGALNACT1
hsa_circRNA_101969	hsa_circ_0041821	0.011283402	0.431741635	1.6567011	chr17	Exonic	NM_032442	NEURL4
hsa_circRNA_000947	hsa_circ_0000947	0.026960269	0.431741635	2.5782047	chr19	Sense overlapping	NM_031485	GRWD1
hsa_circRNA_405717		0.036722435	0.431741635	2.010568	chr19	Intronic	ENST00000301281	UBXN6
hsa_circRNA_002292	hsa_circ_0002292	0.047693181	0.431741635	1.6544175	chr5	Exonic	NM_153013	NADK2
hsa_circRNA_101704	hsa_circ_0037858	0.045400879	0.431741635	2.1431944	chr16	Exonic	NM_004862	LITAF
hsa_circRNA_001063	hsa_circ_0001063	0.042758831	0.431741635	2.315292	chr2	Intergenic		
hsa_circRNA_102509	hsa_circ_0006446	0.034684944	0.431741635	2.2800739	chr19	Exonic	NM_015578	LSM14A
hsa_circRNA_406583		0.045804491	0.431741635	1.6819656	chr5	Sense overlapping	NM_018140	CEP72
hsa_circRNA_102062	hsa_circ_0007990	0.023322108	0.431741635	1.5649695	chr17	Exonic	NM_033419	PGAP3
hsa_circRNA_405781		0.031827149	0.431741635	1.7484564	chr19	Intronic	ENST00000221419	HNRNPL
hsa_circRNA_000746	hsa_circ_0000746	0.001572925	0.431741635	2.0290976	chr17	Antisense	NM_004475	FLOT2
hsa_circRNA_000435	hsa_circ_0000435	0.022928053	0.431741635	1.5484743	chr12	Intronic	ENST00000549893	C12orf75
hsa_circRNA_001714	hsa_circ_0001714	0.010198598	0.431741635	5.0265939	chr7	Exonic	NM_032408	BAZ1B
hsa_circRNA_040206	hsa_circ_0040206	0.036836602	0.431741635	1.5041225	chr16	Exonic	NM_007242	DDX19B
hsa_circRNA_001226	hsa_circ_0001226	0.002126463	0.431741635	2.3072386	chr22	Antisense	NM_002473	MYH9
hsa_circRNA_000134	hsa_circ_0000134	0.049036785	0.431741635	1.7256715	chr1	Antisense	NM_000565	IL6R
hsa_circRNA_087800	hsa_circ_0087800	0.043563969	0.431741635	1.6403757	chr9	Exonic	NM_018376	NIPSNAP3B
hsa_circRNA_400101	hsa_circ_0092328	0.037702213	0.431741635	1.8812764	chr9	Intronic	ENST00000315731	RPL7A
hsa_circRNA_001308	hsa_circ_0001308	0.013850614	0.431741635	3.3527247	chr3	Exonic	NM_003157	NEK4
hsa_circRNA_100659	hsa_circ_0003168	0.049793865	0.431741635	1.5291681	chr10	Exonic	NM_144588	ZFYVE27
hsa_circRNA_404449		0.023726017	0.431741635	1.8863782	chr1	Exonic	NM_032409	PINK1
hsa_circRNA_102774	hsa_circ_0055412	0.044551823	0.431741635	1.5443449	chr2	Exonic	NM_001747	CAPG
hsa_circRNA_102446	hsa_circ_0049356	0.017117814	0.431741635	1.8012178	chr19	Exonic	NM_199141	CARM1
hsa_circRNA_403556		0.00783705	0.431741635	2.0363025	chr6	Exonic	uc010jpp.1	LINC00340
hsa_circRNA_000230	hsa_circ_0000765	0.019997256	0.431741635	1.7827514	chr17	Intronic	ENST00000225916	KAT2A
hsa_circRNA_007326	hsa_circ_0007326	0.046543498	0.431741635	1.9909955	chr14	Exonic	NM_014169	CHMP4A
hsa_circRNA_404807		0.02819908	0.431741635	2.5888983	chr10	Exonic	NM_020682	AS3MT
hsa_circRNA_001389	hsa_circ_0000729	0.027885902	0.431741635	1.5995622	chr16	Intronic	ENST00000268699	GAS8
hsa_circRNA_404818		0.048809072	0.431741635	2.0947754	chr10	Exonic	NM_000274	OAT
hsa_circRNA_001547	hsa_circ_0001874	0.034742413	0.431741635	2.1924449	chr9	Intronic	ENST00000356884	BICD2
hsa_circRNA_001241	hsa_circ_0000508	0.029378216	0.431741635	2.0517604	chr13	Intronic	ENST00000326335	CUL4A
hsa_circRNA_104671	hsa_circ_0001819	0.043208655	0.431741635	1.8112929	chr8	Exonic	NM_015902	UBR5
hsa_circRNA_102442	hsa_circ_0049271	0.044592332	0.431741635	2.611047	chr19	Exonic	NM_012289	KEAP1
hsa_circRNA_003907	hsa_circ_0003907	0.038311645	0.431741635	1.833842	chr13	Intronic	ENST00000319562	FARP1
hsa_circRNA_038516	hsa_circ_0038516	0.039811555	0.431741635	1.7176617	chr16	Exonic	NM_018119	POLR3E
hsa_circRNA_405872		0.031980564	0.431741635	1.6275643	chr2	Exonic	uc002ruu.3	PRKCE
hsa_circRNA_101458	hsa_circ_0034044	0.021127405	0.431741635	1.7423746	chr15	Exonic	uc001ytg.3	HERC2P3
hsa_circRNA_405443		0.003224918	0.431741635	2.1653199	chr16	Intronic	ENST00000342673	NDE1
hsa_circRNA_004077	hsa_circ_0004077	0.037688065	0.431741635	4.1270503	chr16	Exonic	NM_020927	VAT1L
hsa_circRNA_103852	hsa_circ_0072665	0.013650168	0.431741635	2.2677625	chr5	Exonic	NM_197941	ADAMTS6
hsa_circRNA_023461	hsa_circ_0023461	0.000918303	0.431741635	2.3023746	chr11	Exonic	NM_015242	ARAP1
hsa_circRNA_103864	hsa_circ_0005730	0.027626518	0.431741635	2.7818978	chr5	Exonic	NM_001799	CDK7
hsa_circRNA_001653	hsa_circ_0001568	0.016902603	0.431741635	6.1554028	chr6	Intronic	ENST00000344450	DUSP22
hsa_circRNA_001405	hsa_circ_0001167	0.042757718	0.431741635	2.7907614	chr20	Intronic	ENST00000371941	PREX1
hsa_circRNA_043943	hsa_circ_0043943	0.017629978	0.431741635	1.9805323	chr17	Exonic	uc010cyw.1	VAT1
hsa_circRNA_045799	hsa_circ_0045799	0.027973896	0.431741635	1.7012317	chr17	Exonic	NM_022066	UBE2O
hsa_circRNA_406295		0.039669886	0.431741635	1.5046538	chr3	Sense overlapping	NR_109992	SUCLG2-AS1
hsa_circRNA_104484	hsa_circ_0082326	0.035552427	0.431741635	4.3097053	chr7	Exonic	NM_016478	ZC3HC1
hsa_circRNA_100329	hsa_circ_0006352	0.04670856	0.431741635	1.598139	chr1	Exonic	NM_012432	SETDB1
hsa_circRNA_007771	hsa_circ_0007771	0.028286903	0.431741635	1.6641182	chr6	Exonic	NM_032832	LRP11
hsa_circRNA_101491	hsa_circ_0034762	0.039240976	0.431741635	4.4110245	chr15	Exonic	NM_014994	MAPKBP1
hsa_circRNA_020622	hsa_circ_0020622	0.035376567	0.431741635	1.6406534	chr11	Exonic	NM_006435	IFITM2
hsa_circRNA_102481	hsa_circ_0003253	0.016603437	0.431741635	1.7146811	chr19	Exonic	NM_014173	BABAM1
hsa_circRNA_103444	hsa_circ_0008797	0.028562586	0.431741635	2.5886681	chr3	Exonic	NM_002093	GSK3B
hsa_circRNA_104670	hsa_circ_0001818	0.021625832	0.431741635	3.9778781	chr8	Exonic	NM_015902	UBR5
hsa_circRNA_406126		0.023124964	0.431741635	1.757962	chr20	Intronic	ENST00000244070	PPP4R1L
hsa_circRNA_000911	hsa_circ_0001184	0.023141682	0.431741635	1.5147777	chr21	Intronic	ENST00000290219	IFNGR2

FDR: false discover rate; FC: fold change.

**Table 4:** Differentially down-regulated circRNAs in serum exosomes of patients with sepsis.

circRNA	Alias	*P*-value	FDR	FC (abs)	chrom	circRNA_type	best_transcript	GeneSymbol
hsa_circRNA_006750	hsa_circ_0006750	0.037575777	0.431741635	1.5167592	chr10	Exonic	NM_015188	TBC1D12
hsa_circRNA_008289	hsa_circ_0008289	0.007861232	0.431741635	1.5038783	chr6	Exonic	NM_012454	TIAM2
hsa_circRNA_072654	hsa_circ_0072654	0.004150655	0.431741635	3.1968303	chr5	Exonic	NM_005869	CWC27
hsa_circRNA_009554	hsa_circ_0009554	0.044334492	0.431741635	1.5604032	chr1	Exonic	NM_007262	PARK7
hsa_circRNA_030788	hsa_circ_0030788	0.047261899	0.431741635	1.6207698	chr13	Exonic	NM_052867	NALCN
hsa_circRNA_400850		0.036097077	0.431741635	1.650349	chr11	Exonic	NM_016146	TRAPPC4
hsa_circRNA_404459		0.002634492	0.431741635	1.6303638	chr1	Exonic	NM_022778	CEP85
hsa_circRNA_102912	hsa_circ_0058055	0.019467222	0.431741635	1.5068981	chr2	Exonic	NM_000465	BARD1
hsa_circRNA_032891	hsa_circ_0032891	0.031939282	0.431741635	1.5637739	chr14	Exonic	NM_145231	EFCAB11
hsa_circRNA_401829		0.032698187	0.431741635	1.5255687	chr17	Exonic	NM_178509	STXBP4
hsa_circRNA_400511		0.023801242	0.431741635	1.6454873	chr10	Exonic	NM_014142	NUDT5
hsa_circRNA_100726	hsa_circ_0002456	0.025458471	0.431741635	1.5928692	chr10	Exonic	NM_001380	DOCK1
hsa_circRNA_405372		0.039065216	0.431741635	1.5208354	chr15	Sense overlapping	NR_040051	IQCH-AS1
hsa_circRNA_007352	hsa_circ_0007352	0.032409473	0.431741635	4.6954462	chrX	Exonic	NM_005088	AKAP17A
hsa_circRNA_104639	hsa_circ_0084669	0.048813158	0.431741635	1.6255475	chr8	Exonic	NM_024790	CSPP1
hsa_circRNA_406194		0.003824786	0.431741635	2.0373362	chr22	Sense overlapping	NM_013365	GGA1
hsa_circRNA_406445		0.039630011	0.431741635	1.5055446	chr4	Intronic	ENST00000264956	EVC
hsa_circRNA_405571		0.038880048	0.431741635	1.9452313	chr17	Exonic	ENST00000589153	TADA2A
hsa_circRNA_405791		0.016540118	0.431741635	1.5537398	chr19	Exonic	NM_006663	PPP1R13L
hsa_circRNA_104964	hsa_circ_0006502	0.031313741	0.431741635	1.6161558	chr9	Exonic	NM_138778	DPH7
hsa_circRNA_100631	hsa_circ_0006148	0.012110784	0.431741635	2.1672149	chr10	Exonic	NM_144660	SAMD8
hsa_circRNA_405746		0.023710234	0.431741635	1.8437062	chr19	Exonic	NM_032207	C19orf44
hsa_circRNA_101461	hsa_circ_0034072	0.016991154	0.431741635	1.8499723	chr15	Exonic	NM_014608	CYFIP1
hsa_circRNA_063280	hsa_circ_0063280	0.046069864	0.431741635	1.5904218	chr22	Exonic	NM_012407	PICK1
hsa_circRNA_405477		0.02927257	0.431741635	1.7238343	chr16	Intronic	ENST00000264005	LCAT
hsa_circRNA_400042	hsa_circ_0092302	0.025102341	0.431741635	1.5460887	chr19	Intronic	ENST00000325327	LMNB2
hsa_circRNA_040203	hsa_circ_0040203	0.028512125	0.431741635	1.5408761	chr16	Exonic	NM_001605	AARS
hsa_circRNA_076057	hsa_circ_0076057	0.047636875	0.431741635	1.571403	chr6	Exonic	NM_017754	UHRF1BP1
hsa_circRNA_001729	hsa_circ_0000691	0.048652258	0.431741635	1.7920519	chr16	Antisense	NM_014699	ZNF646
hsa_circRNA_004738	hsa_circ_0004738	0.043002838	0.431741635	1.6720137	chr5	Exonic	NM_022897	RANBP17
hsa_circRNA_100559	hsa_circ_0000219	0.014298038	0.431741635	1.5281119	chr10	Exonic	NM_024948	FAM188A
hsa_circRNA_002773	hsa_circ_0002773	0.029869133	0.431741635	1.5045762	chr11	Exonic	NM_002906	RDX
hsa_circRNA_104004	hsa_circ_0074930	0.021445503	0.431741635	1.9530485	chr5	Exonic	NM_003062	SLIT3
hsa_circRNA_100317	hsa_circ_0008390	0.04490215	0.431741635	2.1464941	chr1	Exonic	NM_022359	PDE4DIP
hsa_circRNA_100707	hsa_circ_0020313	0.029667199	0.431741635	1.6620556	chr10	Exonic	NM_022126	LHPP
hsa_circRNA_102461	hsa_circ_0003935	0.013483506	0.431741635	1.5061068	chr19	Exonic	NM_000068	CACNA1A
hsa_circRNA_060123	hsa_circ_0060123	0.028890929	0.431741635	1.5685863	chr20	Exonic	uc002xdn.1	CPNE1
hsa_circRNA_404686		0.012768084	0.431741635	1.9349548	chr1	Exonic	NM_003272	GPR137B
hsa_circRNA_101321	hsa_circ_0002928	0.042321436	0.431741635	1.611344	chr14	Exonic	NM_006109	PRMT5
hsa_circRNA_100536	hsa_circ_0005379	0.041730172	0.431741635	1.9452874	chr10	Exonic	NM_001494	GDI2
hsa_circRNA_400994		0.011009991	0.431741635	1.5005858	chr12	Exonic	uc001syj.2	ZDHHC17
hsa_circRNA_103291	hsa_circ_0006673	0.040743075	0.431741635	1.6483582	chr3	Exonic	NM_025265	TSEN2
hsa_circRNA_102116	hsa_circ_0003258	0.005918665	0.431741635	1.5865527	chr17	Exonic	NM_014897	ZNF652
hsa_circRNA_102950	hsa_circ_0058794	0.043872376	0.431741635	1.7071378	chr2	Exonic	NM_014914	AGAP1
hsa_circRNA_020962	hsa_circ_0020962	0.039359099	0.431741635	1.6353777	chr11	Exonic	uc001mai.1	HBG2
hsa_circRNA_003508	hsa_circ_0003508	0.035035101	0.431741635	1.9070829	chr17	Exonic	NR_036474	GPATCH8
hsa_circRNA_008609	hsa_circ_0008609	0.037088726	0.431741635	1.5778959	chr2	Exonic	NR_028356	MRPL30
hsa_circRNA_100632	hsa_circ_0018905	0.044102213	0.431741635	5.3789756	chr10	Exonic	NM_144660	SAMD8
hsa_circRNA_406475		0.042571045	0.431741635	1.5153569	chr4	Intronic	ENST00000264319	FRYL
hsa_circRNA_401299		0.04743786	0.431741635	1.6724819	chr14	Exonic	NM_145231	EFCAB11
hsa_circRNA_102025	hsa_circ_0007542	0.04630629	0.431741635	1.5477632	chr17	Exonic	NM_000267	NF1
hsa_circRNA_001101	hsa_circ_0001101	0.020138729	0.431741635	1.6929037	chr2	Exonic	NM_020830	WDFY1
hsa_circRNA_012123	hsa_circ_0012123	0.046218436	0.431741635	1.7972517	chr1	Exonic	uc001clf.3	ATP6V0B

FDR: false discover rate; FC: fold change.

### RT-qPCR validation of the differentially expressed circRNAs

**Figure 3:**
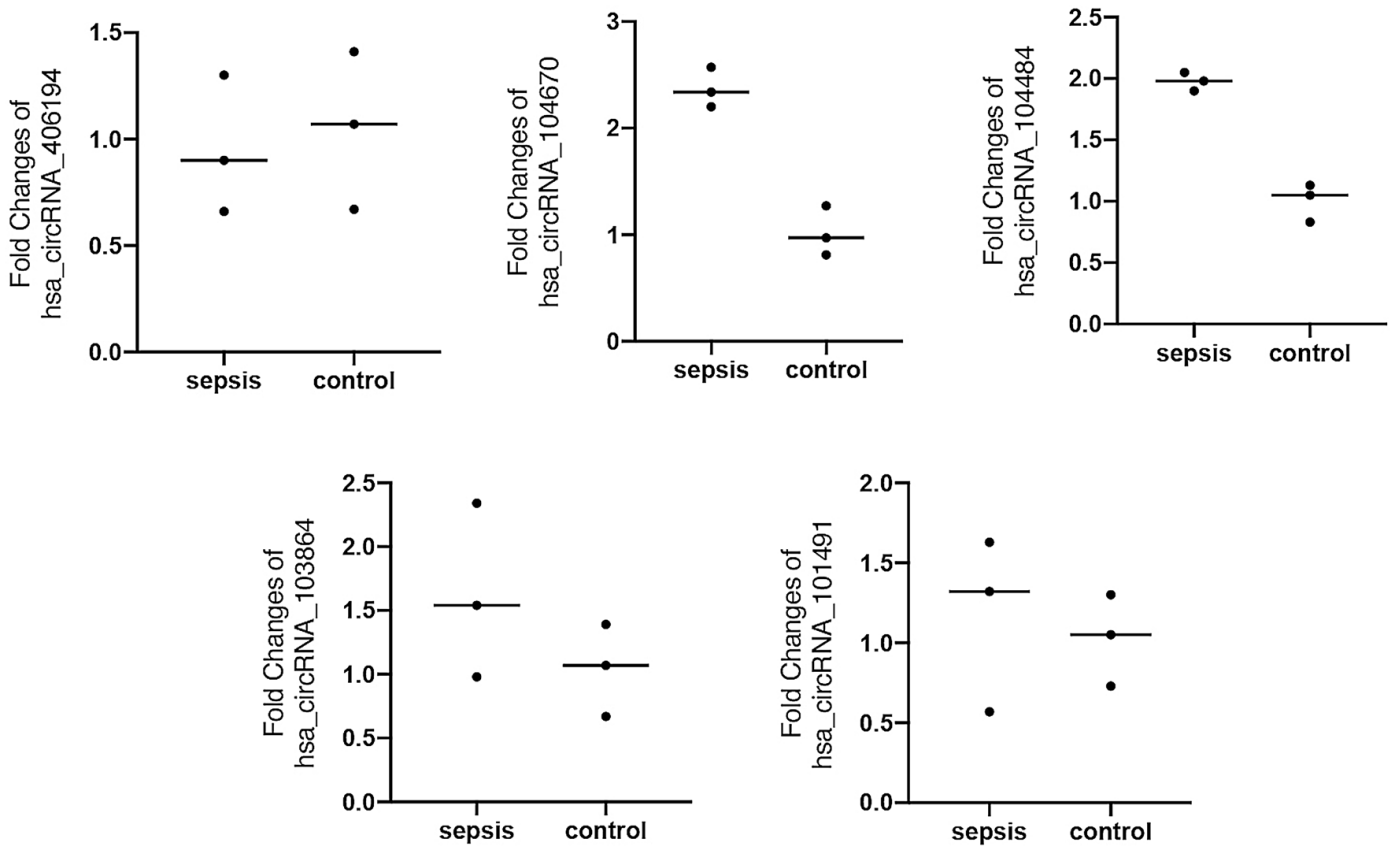
RT-qPCR verification of five circRNAs in microarray samples. The drawings were performed using GraphPad Prism software (version 8.0, https://www.graphpad.com/scientific-software/prism/).

RT-qPCR was used to verify the differentially expressed circRNAs in sepsis. We selected five circRNAs that are most likely to be related to sepsis for verification based on the fold changes in microarray analysis: hsa_circRNA_406194, hsa_circRNA_104670, hsa_circRNA_104484, hsa_circRNA_103864, and hsa_circRNA_101491. Because the microarray analysis may contain false positive results, we first verified in 3 sepsis patients and 3 healthy volunteers that had been tested by microarray to confirm the accurate expression of circRNAs. The expression levels of hsa_circRNA_406194 (0.95 ± 0.32 to 1.05 ± 0.37; *p* = 0.751), hsa_circRNA_104670 (2.37 ± 0.19 to 1.02 ± 0.23; *p* = 0.001), hsa_circRNA_104484 (1.98 ± 0.08 to 1.01 ± 0.15; *p* = 0.001), hsa_circRNA_103864 (1.62 ± 0.68 to 1.04 ± 0.36; *p* = 0.265), and hsa_circRNA_101491 (1.18 ± 0.55 to 1.03 ± 0.28; *p* = 0.699) (Fig. 3). Among these five circRNAs, only hsa_circRNA_104484 and hsa_circRNA_104670 were significantly increased.

**Figure 4:**
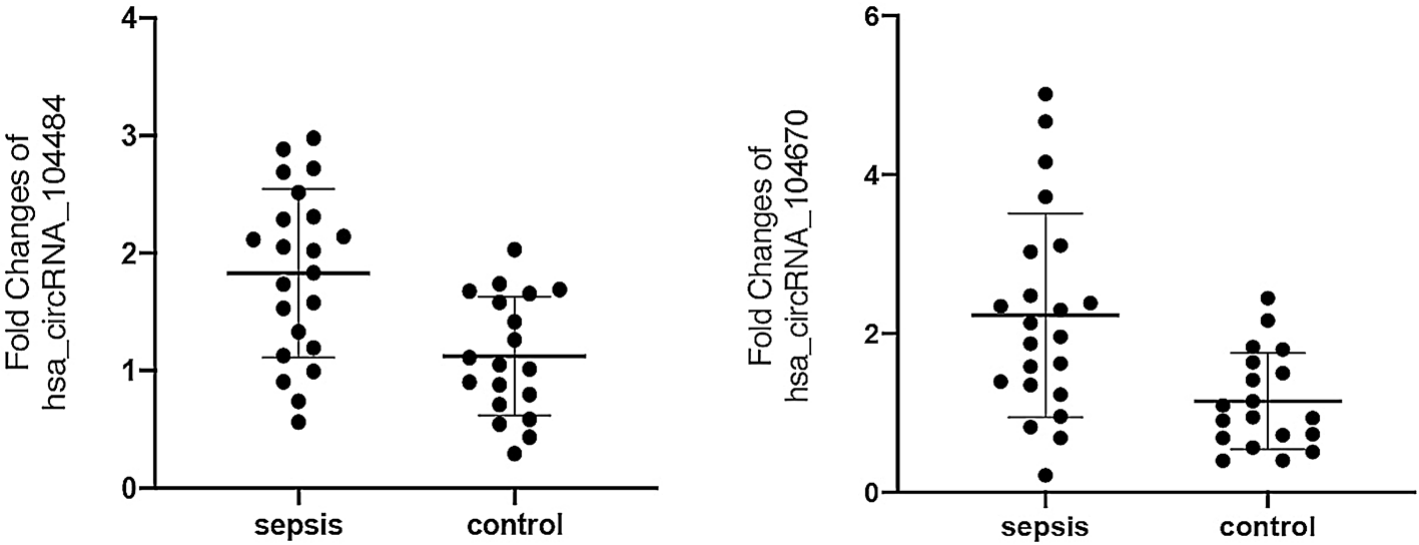
Expression of hsa_circRNA_104484 and hsa_circRNA_104670 in the serum exosomes of 22 patients with sepsis and 19 controls.

We further verified the expression levels of hsa_circRNA_104484 and hsa_circRNA_104670 in the serum exosomes of 22 patients with sepsis and 19 controls collected subsequently. As shown in Fig. 4, the expression of hsa_circRNA_104484 (1.829 ± 0.718 to 1.124 ± 0.506; *p* = 0.005) and hsa_circRNA_104670 (2.045 [1.319–3.049] to 0.948 [0.684–1.639]; *p* = 0.003) in serum exosomes of patients with sepsis increased, and the expression differences were statistically significant, which was consistent with the results of microarray analysis.

### ROC analysis of serum exosomal hsa_circRNA_104484 and hsa_circRNA_104670 in sepsis

**Figure 5:**
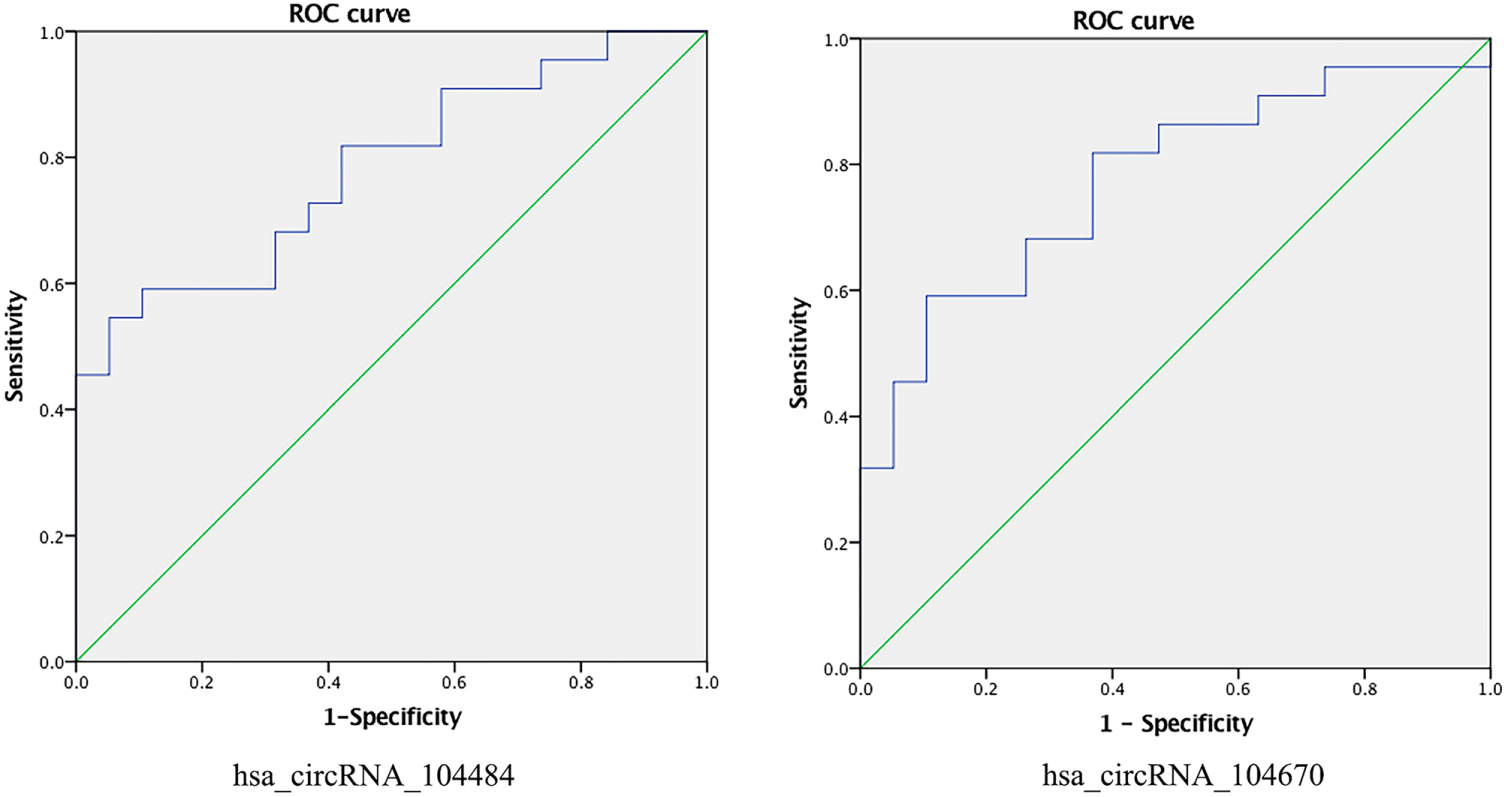
ROC curve for hsa_circRNA_104484 and hsa_circRNA_104670.

The results of qPCR were used to construct the ROC curve to evaluate the diagnostic value of exosomal hsa_circRNA_104484 and hsa_circRNA_104670 in sepsis (Fig. 5). Compared with healthy subjects, the AUC of hsa_circRNA_104484 in sepsis exosomes was 0.782 (95% confidence interval [CI]: 0.643–0.921; *p* < 0.05), the sensitivity and specificity were 0.545 and 0.947, respectively. The highest Youden index was 0.492 and the corresponding optimal cut-off value was 31.901. The AUC of hsa_circRNA_104670 was 0.775 (95% CI: 0.632–0.919; *p* < 0.05), and the sensitivity and specificity were 0.591 and 0.895, respectively. The highest Youden index was 0.486 and the corresponding optimal cut-off value was 1.357. The results indicate that hsa_circRNA_104484 and hsa_circRNA_104670 have a medium diagnostic value and have the potential to be used as diagnostic markers in sepsis.

### Identification of circRNA‐targeting miRNAs and construction of circRNA‐miRNA‐mRNA networks

**Figure 6:**
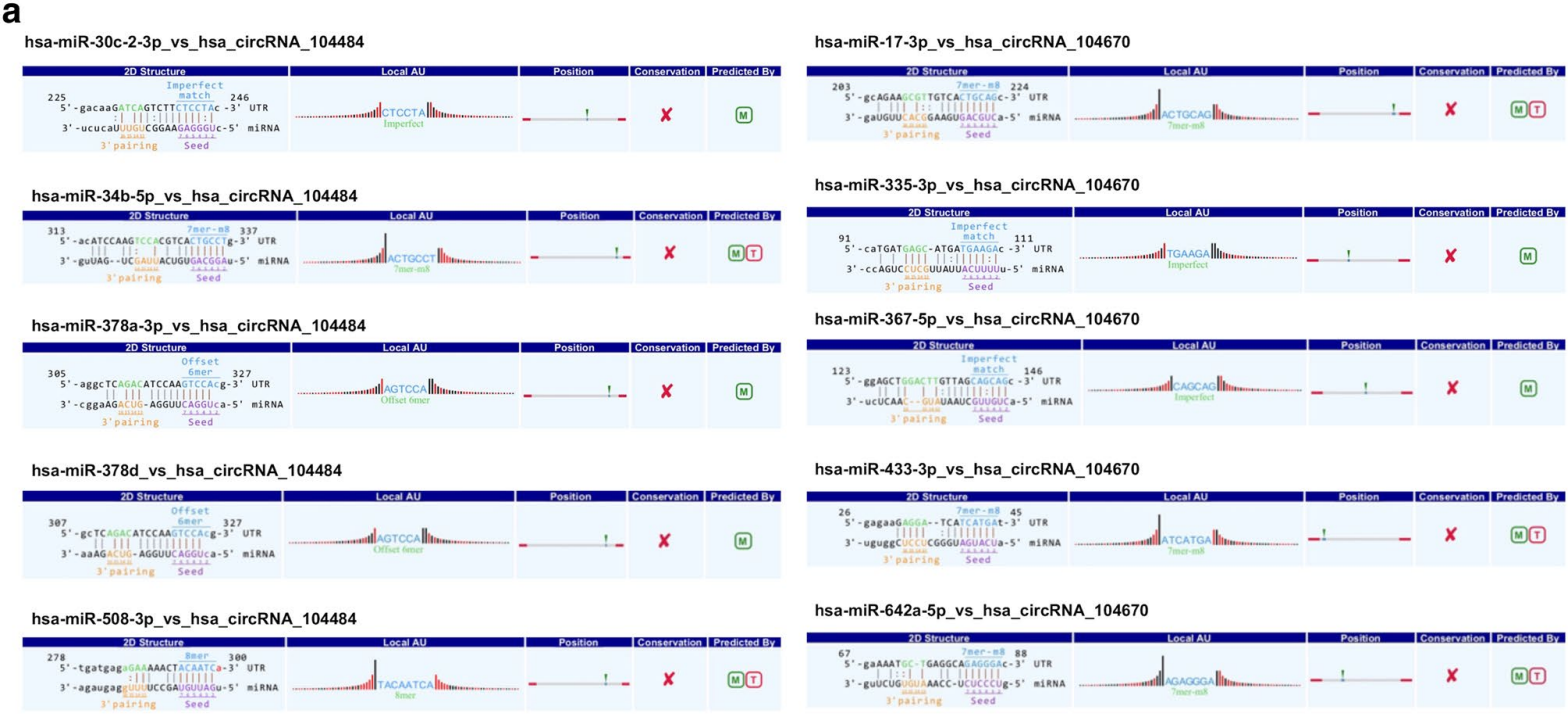

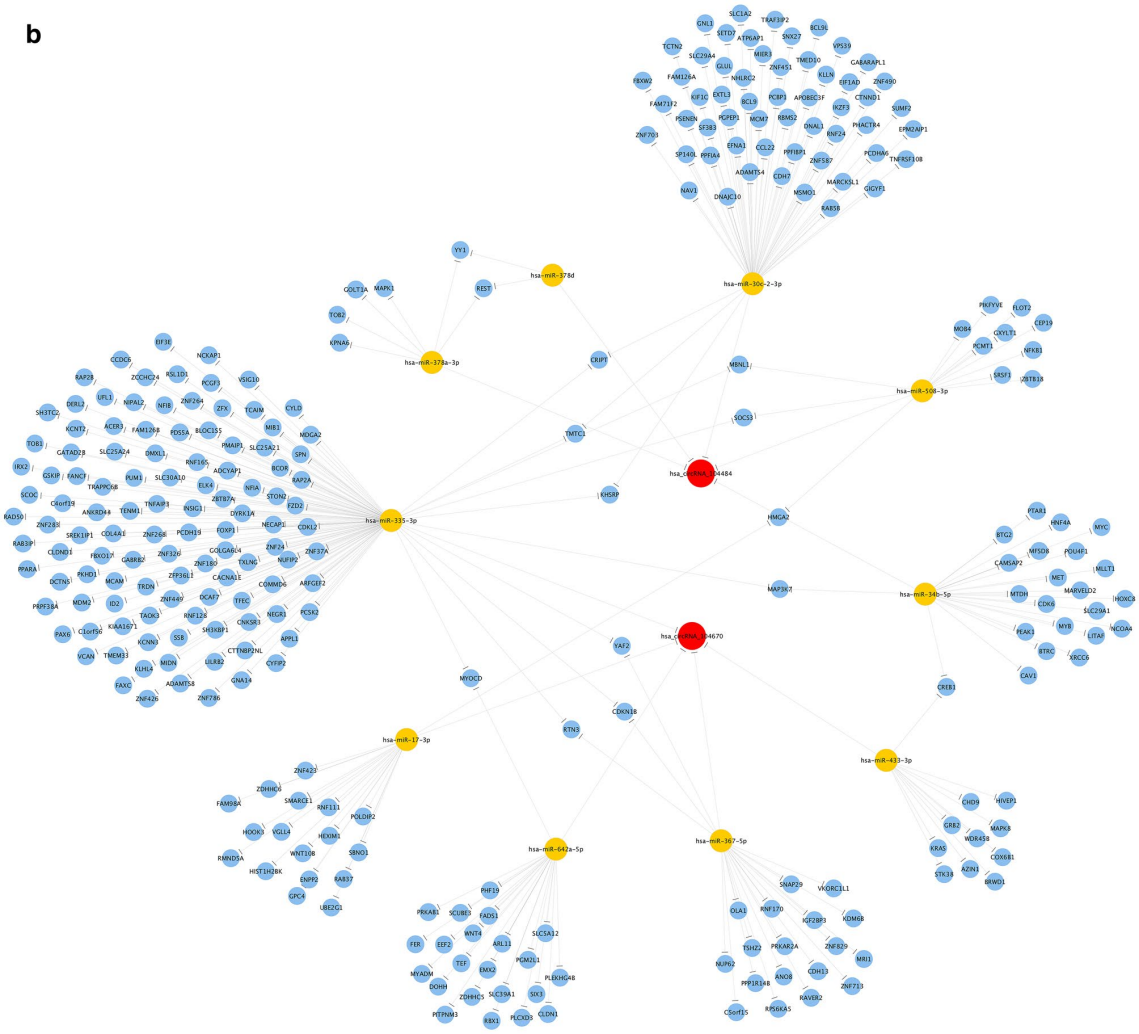
Prediction of circRNA-miRNA-mRNA regulatory relationship. (**a**) Annotation of detailed regulatory relationship between hsa_circRNA_104484, hsa_circRNA_104670 and miRNAs. (**b**) circRNA‐miRNA‐mRNA network established using hsa_circRNA_104484 and hsa_circRNA_104670.

Arraystar microRNA prediction software was used to predict the miRNAs targeted by hsa_circRNA_104484 and hsa_circRNA_104670. The results showed that the miRNAs targeted by hsa_circRNA_104484 were hsa-miR-34b-5p, hsa-miR-508-3p, hsa-miR-378a-3p, hsa-miR-378d, and hsa-miR-30c-2-3p. Further, the miRNAs targeted by hsa_circRNA_104670 were hsa-miR-17-3p, hsa-miR-433-3p, hsa-miR-367-5p, hsa-miR-335-3p, and hsa-miR-642a-5p. The interaction between circRNA-microRNA is annotated in detail, and the results are shown in Fig. 6a. The ceRNA network was used to visually show the relationship between hsa_circRNA_104484 and hsa_circRNA_104670, miRNAs, and target genes (Fig. 6b).

### Prediction of the potential functions of target genes

**Figure 7:**
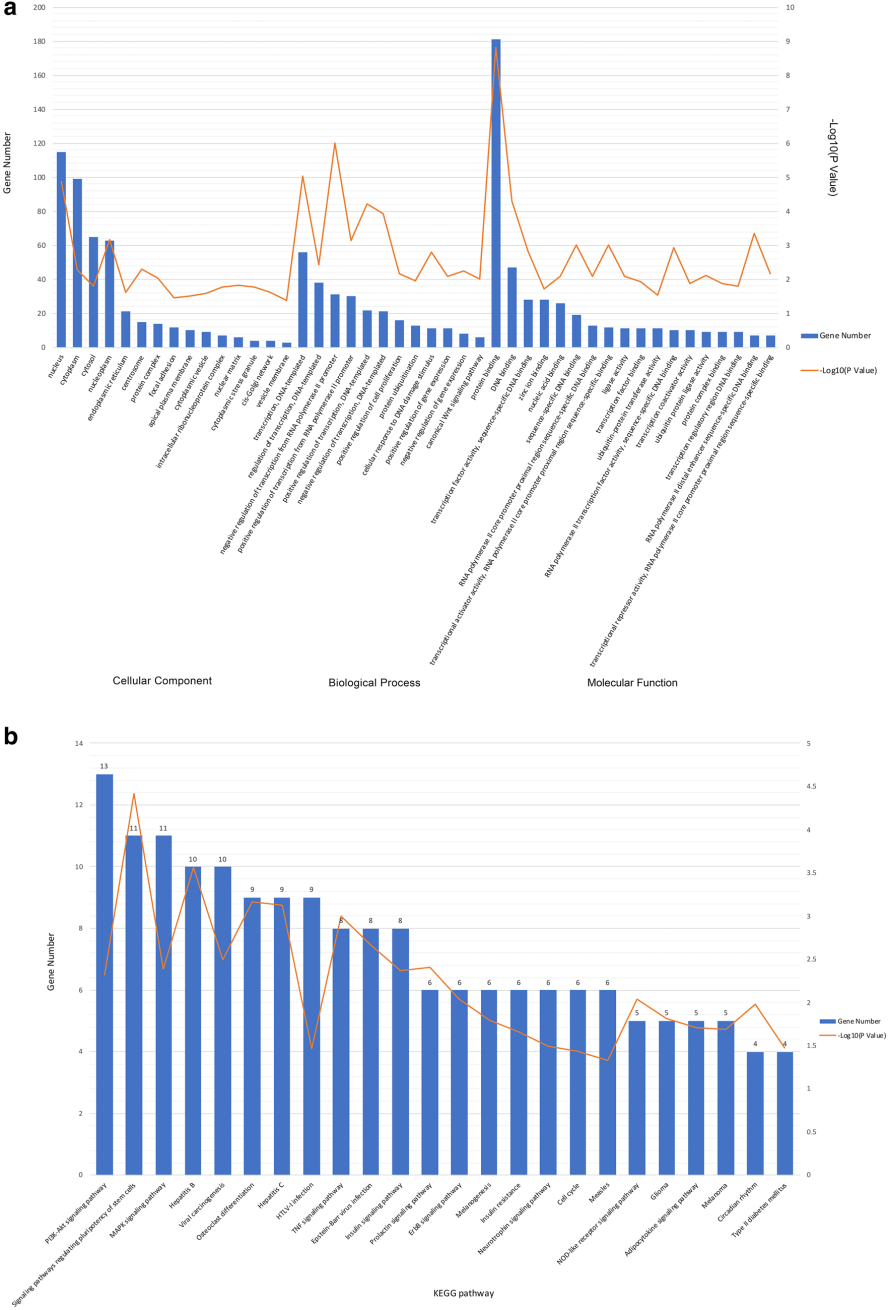
Functional analysis of circRNA. (**a**) Gene Ontology Analysis. (**b**) KEGG pathway Enrichment Analysis. The drawings were performed using Microsoft Excel (version 16.43, https://www.microsoft.com/zh-cn/microsoft-365/excel).

GO analysis results showed that the biological process and molecular functions of target genes were concentrated in several aspects, such as ‘negative regulation of transcription from the RNA polymerase II promoter’, ‘transcription’, ‘positive regulation of transcription’, ‘negative regulation of transcription’, ‘positive regulation of transcription from the RNA polymerase II promoter’, ‘protein binding’, ‘DNA binding’, ‘transcriptional activator activity’, ‘RNA polymerase II transcription factor activity’, ‘transcription factor activity’, and ‘transcriptional repressor activity’ (Fig. 7a). Most of them were related to the transcriptional regulation of gene expression. Therefore, hsa_circRNA_104484 and hsa_circRNA_104670 might participate in the process of sepsis by regulating transcription.

KEGG pathway analysis results show that the target gene-related signalling pathways are the PI3K-Akt signalling pathway, signalling pathways regulating the pluripotency of stem cells, the MAPK signalling pathway, hepatitis B, viral carcinogenesis, osteoclast differentiation, hepatitis C, HTLV-I infection, TNF signalling pathway, and the insulin signalling pathway, among others (Fig. 7b). Among them, the PI3K-Akt signalling pathway^22^, MAPK signalling pathway^23^, and the TNF signalling pathway have been confirmed by several studies to be related to sepsis.

## Discussion

In recent years, despite significant advances in antimicrobial treatment and organ support technologies, sepsis remains the leading cause of death in patients with severe infections^24^. This may be related to the lack of specificity of clinical manifestations, the complexity of pathophysiological processes, and the heterogeneity of sepsis^5^. Unfortunately, despite the continuous exploration of its mechanism, our understanding of it is still far from being sufficient. In fact, there are currently no laboratory testing methods to accurately identify sepsis and there are no individualised therapies to cure it. Therefore, researchers are committed to developing a precision medicine method that aims to classify patients into different types based on transcriptomic signatures and other biological and clinical data, thus providing a molecular basis for precision targeted therapy. Improving the identification and diagnosis of sepsis, exploring its pathogenesis, classification, and individualised therapy can maximise the efficacy and improve prognosis.

In recent years, exosomes have been extensively studied as a new form of intercellular signal transduction. Studies have shown that circRNAs are specifically enriched and stable in exosomes and can be detected in a variety of bodily fluids^17^. This means that exosomal circRNA has the potential to diagnose diseases as a biomarker^5,19^. They are also involved in the pathogenesis of various diseases, such as tumours^25,26^, cardiovascular diseases^27–29^, neurological disorders^30–32^, infections, and immune-related diseases^30,33,34^, indicating that they may be used as targets for precise treatment. To date, the expression and function of exosomal circRNAs in sepsis have not been reported. In order to clarify their regulatory role in the pathophysiology of sepsis, it is necessary to explore the changes in circRNA expression levels in serum exosomes and their regulatory pathways.

By comparing and analysing the results of microarrays, molecules with fold changes > 1.5 and *p* values < 0.05 were considered statistically significant. Then, we selected five circRNA molecules for experimental verification, including hsa_circRNA_101491, hsa_circRNA_103864, hsa_circRNA_104484, hsa_circRNA_104670, and hsa_circRNA_406194. These circRNA molecules were then verified by RT-qPCR among the 3 septic patients and 3 healthy volunteers that had been tested by microarray to determine the reliability of the microarray results. Among these five circRNA molecules, the expression of two circRNA molecules (hsa_circRNA_104484 and hsa_circRNA_104670) were significantly upregulated, consistent with the microarray results, but the other three circRNA molecules (hsa_circRNA_101491, hsa_circRNA_103864, and hsa_circRNA_406194) were not significantly different between the two groups. This indicates that microarray results contain false positives, thus, only differential circRNA molecules qualified by RT-qPCR are considered reliable. We continued to verify hsa_circRNA_104484 and hsa_circRNA_104670 in small clinical samples, and the results are consistent with those of previous studies. To the best of our knowledge, this study is the first report the expression of hsa_circRNA_104484 and hsa_circRNA_104670 in sepsis serum exosomes.

At present, ceRNA is the most common circRNA regulation mechanism. CircRNA targets miRNAs and indirectly regulates the expression of miRNA target genes and plays an important role in the occurrence and development of diseases^35^. Studies have found that circulating miRNAs are differentially expressed in inflammation-related diseases and can target the tumour necrosis factor pathway (TLR/NF-κB signalling pathway), acting as inflammation regulators^36,37^. Therefore, we speculate that circRNA may indirectly regulate the expression of inflammation-related genes by targeting miRNAs in sepsis. The annotation of the circRNA-miRNA regulatory axis and the construction of the ceRNA network showed that five miRNAs and several targeted mRNAs interacted with hsa_circRNA_104484 and hsa_circRNA_104670, respectively.

Among them, hsa_circRNA_104484 is a sponge molecule of hsa-miR-378a-3p/hsa-miR-378d. In recent experimental studies, miR-378 has been found to act directly or indirectly as a regulator of inflammation and participates in the processes of inflammation and immune regulation. Platelet-derived exosomal miR-378a-3p directly targets PDK1, resulting in the inhibition of the Akt/mTOR pathway and promoting the formation of neutrophil extracellular traps (NET) in sepsis^38^. A study by Caserta et al.^36^ showed that miR-378a-3p is differentially expressed in systemic inflammatory response syndrome (SIRS) and correlated with its severity. miR-378a can directly target ZBTB20, which plays a role in cell growth and apoptosis^39^. ZBTB20 is a transcriptional repressor that inhibits the transcription of the IκBα gene and positively regulates the activation of NF-κB, triggering an innate immune response^40,41^. This is consistent with the results of the GO analysis. In addition, miR-378 negatively regulates nuclear respiratory factor-1 (NRF-1), AMP-activated protein kinase γ2 (AMPKγ2), and phosphoinositide 3-kinase (PI3K), inhibits energy metabolism processes, and activates the NF-κB-TNFα pathway, which may be related to SIRS and sepsis^42–44^. Similarly, hsa_circRNA_104670 is a sponge molecule of hsa-miR-17-3p. Jiang and Li et al*.*^45^ found that lipopolysaccharide (LPS) and TNF-α can regulate the expression of miR-17-3p. miR-17-3p directly targets intercellular adhesion molecule 1 (ICAM-1) and inhibits its expression in LPS-induced acute lung injury (ALI)^46^. ICAM-1 is an important inflammatory mediator, and its expression is upregulated in sepsis, which enhances inflammatory cell infiltration and organ damage^47,48^. Therefore, we speculate that hsa_circRNA_104484 and hsa_circRNA_104670 may be involved in the pathogenesis of sepsis.

## Conclusions

Our study compared the differences in the expression levels of circRNAs in serum exosomes between sepsis and healthy people, and initially evaluated the clinical application value of hsa_circRNA_104484 and hsa_circRNA_104670. The results provide a basis for mechanistic research. However, our research sample is relatively small; in the future, the sample size will be enlarged. We will further explore the biological functions of hsa_circRNA_104484 and hsa_circRNA_104670 through cell and animal experiments. Currently, the pathogenesis of sepsis is still unclear. As such, there is no effective therapeutic intervention; the exploration of the circRNA regulatory mechanism in sepsis will have great clinical translation research value.

## Supplementary Information

Supplementary Information.

## Abbreviations

RT-qPCR: Real-time quantitative polymerase chain reaction
ROC: Receiver operating characteristic
GO: Gene ontology
KEGG: Kyoto City Encyclopaedia of Genes and Genomes
circRNAs: Circular RNA
miRNA: MicroRNA
PBS: Phosphate buffer saline
cDNA: Complementary DNA
MF: Molecular functions
BP: Biological pathways
CC: Cellular components
AUC: Area under the ROC curve
TEM: Transmission electron microscopy
CI: Confidence interval
NET: Neutrophil extracellular traps
SIRS: Systemic inflammatory response syndrome
NRF-1: Nuclear respiratory factor-1
AMPKγ2: AMP-activated protein kinase γ2
PI3K: Phosphoinositide 3-kinase
LPS: Lipopolysaccharide
ICAM-1: Intercellular adhesion molecule 1
ALI: Acute lung injury
SOFA: Sequential organ failure assessment

## References

[1] Singer M, Deutschman CS, Seymour CW (2016). The third international consensus definitions for sepsis and septic shock (Sepsis-3). JAMA.

[2] Ferrer R, Martin-Loeches I, Phillips G (2014). Empiric antibiotic treatment reduces mortality in severe sepsis and septic shock from the first hour: results from a guideline-based performance improvement program. Crit. Care Med..

[3] Xu D, Liao S, Li P (2019). Metabolomics coupled with transcriptomics approach deciphering age relevance in sepsis. Aging Dis..

[4] Almansa R, Heredia-Rodriguez M, Gomez-Sanchez E (2015). Transcriptomic correlates of organ failure extent in sepsis. J. Infect..

[5] Leligdowicz A, Matthay MA (2019). Heterogeneity in sepsis: new biological evidence with clinical applications. Crit. Care.

[6] Kalluri R, LeBleu VS (2020). The biology, function, and biomedical applications of exosomes. Science.

[7] Wang X, Gu H, Qin D (2015). Exosomal miR-223 contributes to mesenchymal stem cell-elicited cardioprotection in polymicrobial sepsis. Sci. Rep..

[8] Song Y, Dou H, Li X (2017). Exosomal miR-146a contributes to the enhanced therapeutic efficacy of interleukin-1beta-primed mesenchymal stem cells against sepsis. Stem Cells.

[9] Zhang Y, Liu D, Chen X (2010). Secreted monocytic miR-150 enhances targeted endothelial cell migration. Mol. Cell.

[10] Jeppesen DK, Fenix AM, Franklin JL (2019). Reassessment of exosome composition. Cell.

[11] Ibsen SD, Wright J, Lewis JM (2017). Rapid isolation and detection of exosomes and associated biomarkers from plasma. ACS Nano.

[12] Kourembanas S (2015). Exosomes: vehicles of intercellular signaling, biomarkers, and vectors of cell therapy. Annu. Rev. Physiol..

[13] Kristensen LS, Andersen MS, Stagsted LVW (2019). The biogenesis, biology and characterization of circular RNAs. Nat. Rev. Genet..

[14] Wang PL, Bao Y, Yee MC (2014). Circular RNA is expressed across the eukaryotic tree of life. PLoS ONE.

[15] Lasda E, Parker R (2016). Circular RNAs co-precipitate with extracellular vesicles: a possible mechanism for circRNA clearance. PLoS ONE.

[16] Li Y, Zheng Q, Bao C (2015). Circular RNA is enriched and stable in exosomes: a promising biomarker for cancer diagnosis. Cell Res..

[17] Wang Y, Liu J, Ma J (2019). Exosomal circRNAs: biogenesis, effect and application in human diseases. Mol. Cancer.

[18] Li X, Liu CX, Xue W (2017). Coordinated circRNA biogenesis and function with NF90/NF110 in viral infection. Mol. Cell.

[19] Fanale D, Taverna S, Russo A (2018). Circular RNA in exosomes. Adv. Exp. Med. Biol..

[20] Ritchie ME, Phipson B, Wu D (2015). limma powers differential expression analyses for RNA-sequencing and microarray studies. Nucl. Acids Res..

[21] Kanehisa M, Goto S (2000). KEGG: kyoto encyclopedia of genes and genomes. Nucl. Acids Res..

[22] Xie WJ, Hou G, Wang L (2020). Astaxanthin suppresses lipopolysaccharide-induced myocardial injury by regulating MAPK and PI3K/AKT/mTOR/GSK3β signaling. Mol. Med. Rep..

[23] Wang F, Wang M, Wang J (2020). Maresin1 ameliorates sepsis-associated lung injury by inhibiting the activation of the JAK2/STAT3 and MAPK/NF-κB signaling pathways. Microb. Pathog..

[24] Rudd KE, Johnson SC, Agesa KM (2020). Global, regional, and national sepsis incidence and mortality, 1990–2017: analysis for the Global Burden of Disease Study. Lancet.

[25] Shang A, Gu C, Wang W (2020). Exosomal circPACRGL promotes progression of colorectal cancer via the miR-142-3p/miR-506-3p- TGF-β1 axis. Mol. Cancer.

[26] Huang XY, Huang ZL, Huang J (2020). Exosomal circRNA-100338 promotes hepatocellular carcinoma metastasis via enhancing invasiveness and angiogenesis. J. Exp. Clin. Cancer Res..

[27] Wang W, Wang Y, Piao H (2019). Circular RNAs as potential biomarkers and therapeutics for cardiovascular disease. PeerJ.

[28] Wang Y, Zhao R, Liu W (2019). Exosomal circHIPK3 released from hypoxia-pretreated cardiomyocytes regulates oxidative damage in cardiac microvascular endothelial cells via the miR-29a/IGF-1 pathway. Oxid. Med. Cell Longev.

[29] Han J, Zhang L, Hu L (2020). Circular RNA-expression profiling reveals a potential role of Hsa_circ_0097435 in heart failure via sponging multiple microRNAs. Front. Genet.

[30] He J, Ren M, Li H (2019). Exosomal circular RNA as a biomarker platform for the early diagnosis of immune-mediated demyelinating disease. Front. Genet.

[31] Hosaka T, Yamashita T, Tamaoka A (2019). Extracellular RNAs as biomarkers of sporadic amyotrophic lateral sclerosis and other neurodegenerative diseases. Int. J. Mol. Sci..

[32] Zhao RT, Zhou J, Dong XL (2018). Circular ribonucleic acid expression alteration in exosomes from the brain extracellular space after traumatic brain injury in mice. J. Neurotrauma.

[33] Chen X, Yang T, Wang W (2019). Circular RNAs in immune responses and immune diseases. Theranostics.

[34] Xie R, Zhang Y, Zhang J (2020). The role of circular RNAs in immune-related diseases. Front. Immunol..

[35] Salmena L, Poliseno L, Tay Y (2011). A ceRNA hypothesis: the Rosetta Stone of a hidden RNA language?. Cell.

[36] Caserta S, Mengozzi M, Kern F (1977). Severity of systemic inflammatory response syndrome affects the blood levels of circulating inflammatory-relevant microRNAs. Front. Immunol..

[37] Benz F, Roy S, Trautwein C (2016). Circulating microRNAs as biomarkers for sepsis. Int. J. Mol. Sci..

[38] Jiao Y, Li W, Wang W (2020). Platelet-derived exosomes promote neutrophil extracellular trap formation during septic shock. Crit. Care.

[39] Wang J, Liu ZH, Yu LJ (2019). Long non-coding RNA LINC00641 promotes cell growth and migration through modulating miR-378a/ZBTB20 axis in acute myeloid leukemia. Eur. Rev. Med. Pharmacol. Sci..

[40] Liu X, Zhang P, Bao Y (2013). Zinc finger protein ZBTB20 promotes Toll-like receptor-triggered innate immune responses by repressing IκBα gene transcription. Proc. Natl. Acad. Sci. USA.

[41] Qiu J, Peng P, Xin M (2020). ZBTB20-mediated titanium particle-induced peri-implant osteolysis by promoting macrophage inflammatory responses. Biomater. Sci..

[42] Jeon TI, Park JW, Ahn J (2013). Fisetin protects against hepatosteatosis in mice by inhibiting miR-378. Mol. Nutr. Food Res..

[43] Zhang T, Hu J, Wang X (2019). MicroRNA-378 promotes hepatic inflammation and fibrosis via modulation of the NF-κB-TNFα pathway. J. Hepatol..

[44] Liu W, Cao H, Ye C (2014). Hepatic miR-378 targets p110alpha and controls glucose and lipid homeostasis by modulating hepatic insulin signalling. Nat. Commun..

[45] Jiang X, Li N (2011). Induction of MiR-17-3p and MiR-106a [corrected] by TNFα and LPS. Cell. Biochem. Funct..

[46] Suárez Y, Wang C, Manes TD (2010). Cutting edge: TNF-induced microRNAs regulate TNF-induced expression of E-selectin and intercellular adhesion molecule-1 on human endothelial cells: feedback control of inflammation. J. Immunol..

[47] Laudes IJ, Guo RF, Riedemann NC (2004). Disturbed homeostasis of lung intercellular adhesion molecule-1 and vascular cell adhesion molecule-1 during sepsis. Am. J. Pathol..

[48] Hildebrand F, Pape HC, Harwood P (2005). Role of adhesion molecule ICAM in the pathogenesis of polymicrobial sepsis. Exp. Toxicol. Pathol..

