# Supplementary material for: Exosomal hsa_circRNA_104484 and hsa_circRNA_104670 may serve as potential novel biomarkers and therapeutic targets for sepsis
Source: Sci Rep. 2021 Jul 8;11:14141. doi: 10.1038/s41598-021-93246-0 (PMC8266806; doi:10.1038/s41598-021-93246-0)
Supplement: Supplementary file 1 — Supplementary Information. [file 41598_2021_93246_MOESM1_ESM.pdf]

Supplementary Information

Exosomal hsa_circRNA_104484 and hsa_circRNA_104670 may serve as potential novel biomarkers and therapeutic targets for sepsis

Authors: Chang Tian, Jiaying Liu, Xin Di, Shan Cong, Min Zhao and Ke Wang

Department of Respiratory Medicine, The Second Hospital of Jilin University, Changchun, Jilin, China

Supplementary information

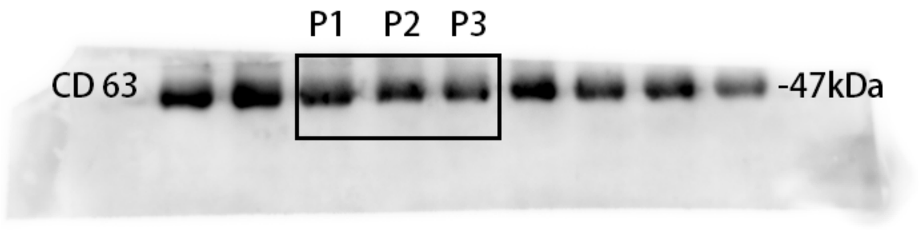

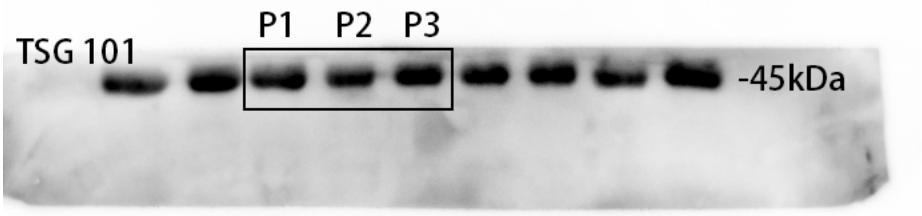

Figure S1. Uncropped images of Figure 1b
